# PERCEPTIONS OF THE ROLE PLAYED BY LIVING ALONE IN PROVIDING SERVICES TO PATIENTS WITH COGNITIVE IMPAIRMENT IN THE US

**DOI:** 10.1093/geroni/igad104.3519

**Published:** 2023-12-21

**Authors:** Elena Portacolone, Julene Johnson, Ashwin Kotwal, Robyn Stone

**Affiliations:** University of California, Berkeley, California, United States; University of California, San Francisco, San Francisco, California, United States; University of California, San Francisco, San Francisco, California, United States; LeadingAge, Washington, District of Columbia, United States

## Abstract

Given the estimated 4.3 million older adults from diverse racial/ethnic backgrounds who are living alone with cognitive impairment in the US, the objective of this study was to identify the potential moderating role of living alone in the use of healthcare and social services for patients with cognitive impairment by eliciting providers’ perceptions of caring for diverse patients with cognitive impairment living alone, in comparison to counterparts living with others. This study used semi-structured interviews conducted between March 2021 and June 2022 with purposively sampled providers of services to patients with cognitive impairment in MI, CA, and TX. An inductive content analysis was used to analyze the data. The majority of the 76 interviewed providers cared for racially/ethnically diverse patientt. Providers represented 20 professions. Providers elucidated specific factors that made serving people living alone with cognitive impairment (PLACI) more challenging, as well as specific factors that increased their concerns when serving PLACI. Providers also elucidated reasons for systematic unmet needs of PLACI for essential healthcare and social services. The vast majority of providers underscored the critical, yet understudied, role of living alone in moderating access to essential healthcare and social services among patients with cognitive impairment. Findings suggest that living arrangement are a social determinant to health among patients with cognitive impairment because PLACI are more likely to experience gaps in services because they are more challenging to serve than counterparts living with others and the healthcare system is not equipped to address these challenges.

